# How Iranian primary health care policies influenced equity: a historical critical analysis from policymakers’ perspectives

**DOI:** 10.1186/s12913-025-12736-3

**Published:** 2025-11-25

**Authors:** Tayebeh Moradi, Negar Yousefzadeh, Efat Mohamadi, Mohammad Mehdi Kiani, Aidin Aryankhesal, Alireza Olyaee Manesh, Amirhossein Takian

**Affiliations:** 1 Department of Health Services Management, School of Health Management and Information Sciences, Iran University of Medical Sciences, Tehran, Iran; 2 National Centre for Health Insurance Research, Tehran, Iran; 3 NIHR Innovation Observatory, Population Health Sciences Institute, Newcastle Medical University, Newcastle Upon Tyne, UK; 4 Health Equity Research Centre (HERC), Tehran University of Medical Sciences (TUMS), Tehran, Iran; 5 Department of Health Management and Economics, School of Public Health, Tehran University of Medical Sciences, Tehran, Iran; 6 School of Health Sciences, Faculty of Medicine and Health Sciences, University of East Anglia, Norwich, England UK; 7 National Institute of Health Research, Tehran University of Medical Sciences, Tehran, Iran; 8 Department of Global Health and Public Policy, School of Public Health, Tehran, Iran

**Keywords:** Primary health care, Health equity, Health network, Referral system, Delivery of health care, Health policy

## Abstract

**Introduction:**

This study aimed to explore how Iranian primary health care (PHC) policies have influenced equity in Iran’s health system over the last 50 years, from the perspectives of policymakers.

**Methodology:**

This qualitative research was conducted between 2019 and 2020. Following the identification, screening, and selection of the most relevant PHC policies through document analysis and expert consultation, 30 semi-structured interviews were conducted with various experts in Iran’s health system. The logical relationships among the data were analysed using the health policy triangle and thematic content analysis, facilitated by MaxQDA software.

**Findings:**

Among the 28 identified PHC policies, five policy groups were recognised as the most significant in relation to health equity. These policies, particularly the PHC and District-level Health Networks (DHNs), have been instrumental in promoting social participation, intersectoral collaboration, and social equity, particularly in addressing acute and communicable diseases. The policies have also ensured equal access to basic health services, especially in rural areas, and have significantly impacted the delivery of care to the population across Iran when acute and communicable diseases were the primary health burden. However, in recent decades, the PHC and DHNs have struggled to keep pace with the dynamic societal changes, shifting disease patterns, and technological advancements.

**Discussion and conclusion:**

While the policies have been successful in providing equitable care for acute and communicable diseases, improvements are required to address the rising burden of non-communicable diseases (NCDs). The integration of NCD care into Iran’s PHC and DHN requires a cultural shift towards preventive health and lifestyle changes. Political will and support from both the government and healthcare policymakers are essential to overcome barriers such as inherent conflicts of interest.

**Supplementary Information:**

The online version contains supplementary material available at 10.1186/s12913-025-12736-3.

## Introduction

Primary Health Care (PHC) was first highlighted by the World Health Organization (WHO) in the Alma-Ata Declaration of September 1978, under the slogan “Health for All by the Year 2000“ [1]. Since then, many countries have incorporated PHC into their health agendas, prioritising it as a central component of their health systems [2]. PHC serves as the first point of contact within the healthcare system, delivering preventive, promotive, curative, and rehabilitative services and plays a pivotal role in promoting equity as evidence suggests [3–5]. However, despite over 40 years of advancements in healthcare, health inequities and disparities in health outcomes persist globally [2].

For PHC to effectively promote equity, it must be carefully designed and deeply integrated into the health system’s infrastructure and the culture of society [6]. Several strategies, such as strengthening intersectoral collaboration, can enable PHC and health networks to address and reduce health inequities [7, 8]. Additionally, policy measures such as ensuring universal access, adopting a horizontal, community-oriented approach, providing adequate training and employing appropriate healthcare personnel, integrating PHC with other services in the regional health system, and organising these services within a comprehensive intersectoral network at the national, provincial, and local levels can significantly contribute to reducing health inequities [9]. Various countries around the world have developed different PHC models to achieve equity; however, the Iranian PHC model was once considered one of the most successful [10].

### PHC history in Iran

**Fig. 1:**
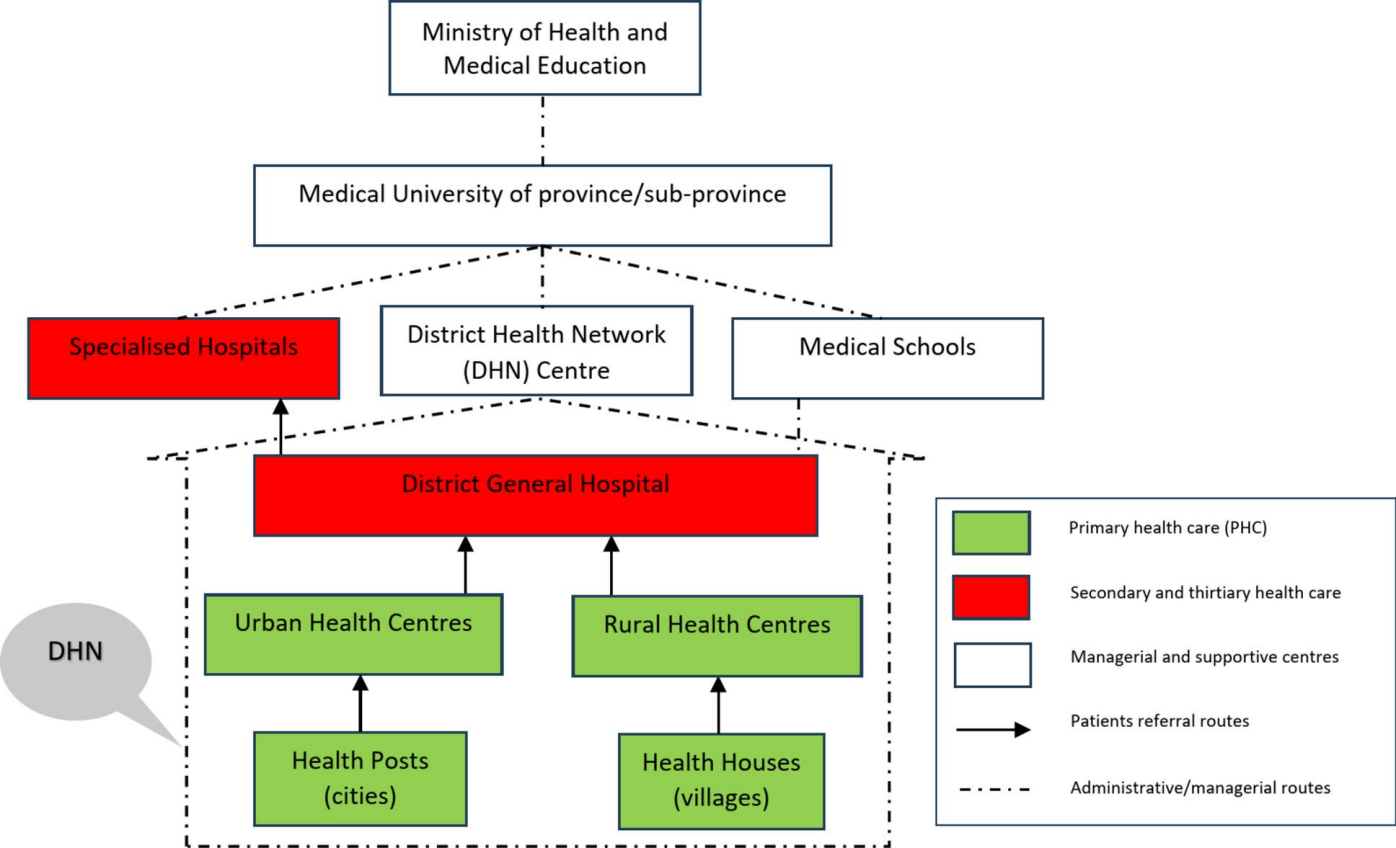
The formal structure of District-level Health Network (DHN) and its position in Iran’s health system

In 1984, Iran officially took a major step to strengthen primary health care (PHC) by establishing a “district-level health network” or shortly District Health Network (DHN) and referral system [11]. A comprehensive plan for this district-level health network was designed at the national level, structured as an infrastructure in accordance with World Health Organization (WHO) norms and standards [12] (see Fig. 1). The goal of establishing DHNs was to shape a gatekeeping system of care delivery including primary and secondary care in each district which needs referral orders for patients to be admitted at higher levels of care delivery. Each DHN comprised Health Posts in cities and Health Houses in villages to provide elementary primary care by skilled staff, rural and urban health centres to provide primary care by general practitioners, and a district general hospital across each district. If more advanced care was necessary, patients can be referred to specialised hospitals. The DHNs were affiliated to provincial or sub-provincial medical universities that were in charge of managing and supporting the DHNs, as well as supporting hospitals and medical schools in their catchment areas [13].

The DHNs and their referral routes were designed to ensure that individuals anywhere in Iran could access health houses or health posts within an hour’s walking distance. The care provided through DHNs was free of charge, provided that patients were referred through the referral orders from a lower level of care delivery centres. Reimbursement for services was facilitated through Iran’s two primary public insurance organisations, Health Services Insurance and Social Security Insurance, which had compulsory membership for the population.

For several years, Iran gained international recognition as a pioneer in the implementation of a comprehensive PHC through its DHNs [14]. An analysis of DHNs and PHC in Iran reveals notable achievements and positive health outcomes, such as reductions in maternal and child mortality and a significant decline in infectious diseases [10]. Nevertheless, parallel and even conflicting interventions and measures have posed challenges to equity within Iran’s health system [15, 16].

While Iran’s PHC and DHNs effectively met health needs in the 1980s, the existing system has struggled in recent years to respond to contemporary health demands and manage health emergencies. This decline in responsiveness is largely due to shifts in the burden of disease and changes in health service demand patterns [10, 15]. Such limitations may have a detrimental impact on PHC equity [3].

While statistics related to infant, neonatal, and maternal mortality rates and some communicable diseases have shown great decrease and higher equity since the establishment of DHNs [17], four major classes of non-communicable diseases (NCDs) including cancer, cardiovascular diseases, asthma and chronic obstructive pulmonary disease (COPD), and diabetes, indicate significant disparities in age-standardised mortality rates across the country’s provinces and projections for 2030 also suggest a continuing disparity in mortality rates for these disease classes [18], highlighting inequity in key primary health indicators across the country. Evidence further suggests substantial inequities in the distribution of healthcare facilities in Iran, particularly about hospitals and those that should deal with NCDs at their advanced stages [19].

Additionally, the Health System Transformation Plan, launched in May 2014, which originally aimed to improve healthcare access, reduce out-of-pocket expenses, and enhance the quality of healthcare services by expanding insurance coverage, strengthening primary healthcare, and investing in hospital infrastructure and human resources, has widened the gap between PHC and secondary care due to shifting budgets towards secondary and tertiary care in the cost of minimizing primary care’s share [20]. As a result, DHSs and consequently the PHC has effectively lost their central role in the provision of health services in Iran.

Given the complexities of assessing health equity through the lens of service provision, particularly PHC, reaching a comprehensive consensus in this area remains elusive [8]. Accordingly, this study, by analysing the policy documents and stakeholders’ viewpoints mainly through a qualitative approach, aims to explore how Iranian PHC policies have influenced equity in the health system over the past 50 years. The findings offer valuable insights for policy reforms aimed at achieving equity in PHC globally.

## Methodology

We conducted a mixed-methods research approach (nested concurrent design) [21], with the main qualitative design and a small quantitative section. was employed from 2019 to 2020, utilising both documentary analysis and the health policy triangle framework to explore and explain the complexity of the issue. The study was conducted in three main phases: document analysis, policy selection, and policy analysis, detailed as follows.

### Phase I: document analysis

In the initial phase, a comprehensive document analysis was carried out between October and December 2019, focusing on all regulations and policies concerning Iran’s PHC, DHNs, and referral system. The aim was to assess the impact of these policies on health equity over the past 50 years. Two researchers independently identified relevant documents through searches on the websites of the Iranian Legislative Assembly and associated organisations. The documents examined included the Constitution, the Universal Medical Insurance Act, the Law on the Comprehensive Welfare and Social Security Organisational Structure, the Law on General Plans and National Development Plans (NDPs), the Health Transformation Plan, the Council of Ministers’ approvals, permanent decrees under the Law on National Development Plans (NDPs), the 20-year Perspective Document for Iran (to 2025), and the National Land Use Policy.

The inclusion criteria for document selection were as follows:

1. *Level or type of document*: Documents were categorised into four levels—vision, strategy, policy, or action. Only those classified as policies were selected, as visions lacked policy-specific details, strategies often did not specify a single executive body, and actions were considered subsets of policies. Policies could pertain to the general sector, health sector, or health system; in this study, only health system policies were considered, with unrelated policies excluded following a consensus during a brainstorming session among the researchers.
2. *Implementation*: Only fully implemented policies were included. Policies that existed only on paper were excluded from the study. The policies were tracked through available online documents to define if they were implemented.
3. *Goals*: Only policies with explicit objectives related to promoting or expanding equity in at least one of three areas—access, financial resource allocation, or policy intervention outcomes—were included.
4. *Scale*: Only policies at national scale, rather than regional or provincial scales, were included. In general, health policy-making in Iran primarily occurs at the central or federal government level, with limited authority for provinces to enact changes.
5. *Timing*: Due to the study’s historical focus, policies from the past 50 years were examined.

A data extraction form was developed to collect information from the selected documents. Two researchers (TM and NY) independently extracted the data, which was summarised in an Excel file. The columns included policy characteristics such as title, source, date of enforcement, enacting body, type of document, implementation status, goals, scale, timeline, and a content summary. Disagreements were resolved through discussions with a senior researcher (AA).

### Phase II: policy selection

Following the review of 23 upstream documents and laws related to PHC, the referral system, and the DHNs in Iran, a screening process was undertaken to identify policies with the greatest impact on health equity. We invited 30 experts in the field of health policy to conduct the survey, each with a minimum of 10 years’ experience and relevant education, as well as familiarity with PHC, DHNs, or referral systems and health equity (see Table 1). Experts were asked to rate the policies’ influence on a 5-point Likert scale (1 = least influence, 5 = greatest influence), as well as one open-ended question for any relevant missing policy in the list. Data collection took one month, with weekly follow-ups (up to two times). In total, 23 experts responded (response rate: ~ 77%).

**Table 1:** Participants’ characteristics in policy selection (phase II), and policy analysis (phase III)

Characteristics		Frequency (%)	
		Phase II (*n* = 23)	Phase III (*n* = 30)
Age (years)	31–40	2 (8.7)	2 (6.7)
	41–50	6 (26.1)	8 (26.7)
	51–60	9 (39.1)	15 (50)
	60<	6 (26.1)	5 (16.7)
Gender	Female	5 (21.7)	8 (26.7)
	Male	18 (78.3)	22 (73.3)
Work experience (years)	5–10	1 (4.3)	2 (6.7)
	11–15	7 (30.4)	6 (20)
	16–20	9 (39.1)	7 (23.3)
	21≤	6 (26.1)	15 (50)
Education degree	Master’s degree	7 (30.4)	4 (13.3)
	Professional doctorate, specialty and subspecialty, and higher	16 (69.6)	26 (86.7)
Organizational affiliation	Ministry of Health and Medical Education	7 (30.4)	11 (36.7)
	Universities of Medical Sciences	8 (34.8)	8 (26.7)
	Social Security Organization	5 (21.7)	3 (10)
	Iran Health Insurance Organization	3 (13)	8 (26.7)

### Phase III: policy analysis

In the final phase, a snowball sampling method was used to conduct semi-structured interviews with open-ended questions. Overall, 30 experts were interviewed, and data saturation was achieved with these interviews. This group included ministerial managers, planners, policymakers, and university professors (see Table 1).

The selection criteria for participants were:

- Relevant academic qualifications in fields such as general medicine, family medicine, public health, family health, health service management, health policy, or health economics (at the master’s or doctoral level).
- A minimum of five years’ experience in policymaking, decision-making, or planning within areas related to PHC, family medicine, the referral system, the DHNs, and health equity.
- Involvement in scientific research related to these areas.

The interviews were conducted by the primary researchers (NY, TM, and AA), who were well-versed in interview techniques and knowledgeable about the research topic. Face-to-face interviews took place at the participants’ workplaces, with each session lasting between 31 and 58 min, averaging 43 min. Prior to each interview, the researchers provided an overview of the study’s objectives, after which informed consent was obtained. Participants were assured of the confidentiality of their information. The interview guide is provided in the Appendix.

All interviews were recorded and transcribed verbatim, and the transcripts were sent to participants for validation. The data were coded using MaxQDA 10 software. Two researchers (NY and TM) independently coded the first two interviews, and any discrepancies were resolved through discussions with the senior researcher (AA). The remainder of the coding process was carried out collaboratively by the two lead researchers.

Using Walt and Gilson’s [22] framework, the logical relationships between the data were analysed, while thematic content analysis [23] was employed to identify and group themes and sub-themes. The outcomes of each policy, including reasons for its success or failure and any policy recommendations proposed by the researchers, were also examined. To ensure data credibility, two qualitative research specialists (AA and AT) reviewed and monitored the coding process.

## Results

In the first phase of the study, a document analysis identified 28 groups of PHC policies. Following the survey conducted in the second phase, five groups of policies were selected as having the greatest influence on health equity (see Table 2). These selected policies were subsequently analysed under six main themes, as outlined in Table 3 and discussed in the following sections.

**Table 2:** The selected policies related to Iran’s PHC aiming to reduce health inequity

Policy address	Starting year	Summary of the policy content
Clause 8 of General Health Policies	2012	Increasing and improving the quality and safety of comprehensive and integrated health care services with focus on equity, accountability, transparent information, effectiveness, efficiency, and productivity in the form of a health network in accordance with the service provision at regionalisation and referral system
Article 73 of National Land Use Policy	2004	Fair access to health services in the country through the planning of the health network based on regionalized service provision
Note 94 of the second 5-year National Development Plan	1994	Completing the facilities, equipment, and human forces of the country’s healthcare network with the government’s public revenues in order of priority: health houses, rural health centers, health bases, and urban health centers
Article 11 of the Universal Medical Insurance Act	1994	Provision of free health services through the health network of the Ministry of Health and Medical Education
Clause 6 under Section B, Part 1 of the second 5-year National Development Plan	1984	Fulfilling the minimum basic needs of the people, including food to the extent of biological needs, general health education, and primary health care for the public with an emphasis on deprived and rural areas of the country, and providing environmental sanitation

**Table 3:** Categories and themes related concerning PHC and health inequity

Categories	Theme	*N*. of sub-themes	*N* of codes
Policy content	Look at achievement of equity in people’s access to PHC	4	396
	Active delivery of care to the covered population with the priority of disease prevention and education of health principles	3	
Context	Legal and political dimension	2	457
	Economic dimension	2	
	Socio-cultural dimension	4	
	Technical dimension	3	
Process	Putting on the agenda and formulating policies	3	471
	Policy implementation	4	
	Policy evaluation	3	
Actors	Opposing actors	2	153
	Consenting actors	5	
Success factors	Appropriate process design for provision levels, service integration, and service delivery through the referral system	2	268
	Powerful laws to implement PHC and health network	2	
Failure factors	Poor implementation of policies and weakness in implementation of the referral system along with establishment of PHC	3	372
	Lack of participation and common belief among policy makers, executives, and people, and conflict of interests in network system	5	
	Transition from communicable to non-communicable diseases and lack of preparedness plans	2	

### Policy content

The analysis of the policy content concerning PHC, the DHNs, and the referral system is detailed below.

#### Equity in access to primary health care

In 1984, District-level Health Networks (DHNs) were established in Iran, prioritising rural, deprived, and remote areas, and encouraging public participation in health-related matters. This initiative was driven by the Ministry’s proactive stance on primary health care, which aimed to foster social equity through the selection and training of local personnel. The DHNs were designed around multiple levels of healthcare provision, with the referral system serving as a guiding framework.

One participant remarked: P 12: *“Policymakers recognised that equitable access to services in rural areas could be achieved through the establishment of PHC and the DHNs. In fact*,* during the development of these policies*,* equity—particularly equitable access and the prioritisation of PHC—was extensively discussed in the pursuit of equity.”*

#### Active service delivery, with priority of preventive care and education

Several interviewees emphasised that health is a fundamental human right, and its provision should be a priority for all governments, particularly from an equity-oriented perspective. However, they also noted that active service delivery, which includes providing care at rural population’s home if they are unable to visit clinics, could not be achieved through governance alone. Educating the public, improving health literacy, and encouraging lifestyle changes, particularly those focused on preventing controllable health issues, were deemed essential components of the system. The PHC and DHNs, which were established to facilitate the distribution of healthcare resources based on the principles of vertical and horizontal equity, positioned health houses as the initial point of contact between individuals and the health system.

One participant elaborated: P 4: *“The PHC and DHNs mean that we consider all regions of the country. This ensures that all people*,* even in the most remote geographical areas*,* receive active healthcare services—not merely for treatment*,* but primarily for prevention. It signifies that individuals do not need to see a specialist for common illnesses like a cold*,* but instead follow a reasonable*,* predetermined care pathway.”*

### Policy context

The policy context addresses legal, political, economic, sociocultural, and technical dimensions. The interviewees raised the following points regarding the PHC and DHNs in their pursuit of achieving equity:

#### Powerful supporting laws for the implementation of PHC and DHNs

Supporting laws are crucial for reinforcing PHC and DHNs, providing executives with the authority to implement policies effectively. Numerous laws have been enacted by various legal authorities, including key national documents such as the Constitution, national development plans, the Public Insurance Law, and the approval letters of the Council of Ministers.

As one participant explained: P 1: *“Since the 1980 s and even earlier*,* the PHC and the establishment of the DHNs have been enshrined in the law. In fact*,* there was a common message among all major stakeholders: they sought to implement fundamental measures to ensure access to healthcare. This approach to healthcare provision is both equitable and effective.”*

#### Providing services at more reasonable costs

From an economic perspective, geographic access to services was influenced by two key factors: distance and route. Policies aimed to ensure that no individual in any geographical or climatic situation would have to travel more than one hour on foot to reach a service delivery centre, and the location should be along their natural travel route. The policy also reduced the cost of travel for basic health services. Economically, it was essential that services remained affordable for individuals, families, and communities. To reduce costs, programmes included the use of non-physician staff, service provision at various levels of healthcare, and even the free provision of some medicines.

One respondent highlighted: P 3: *“One of the primary goals of these structures for delivering PHC was to promote equity. By prioritising rural areas*,* people received essential services through direct government insurance. In my opinion*,* one of the most important aspects of equity is ensuring economic access to healthcare.”*

#### Attracting social participation by training local health workers

PHC and DHNs policies recognised the importance of community-oriented workforce training. This involved developing communication skills to teach basic healthcare principles to populations in diverse geographical areas. Local health workers, familiar with regional cultural and social values, were seen as essential to institutionalising primary care principles and promoting lifestyle changes. Social participation and health movement initiatives required health workers from within the community, capable of fostering new attitudes and behaviours. In line with this approach, the national health-worker programme in Iran commenced soon after the Alma-Ata Conference in 1979, with the first health-worker training centre established between 1984 and 1986.

As one interviewee commented: P 4: *“We said that someone from within the community should educate the people. For example*,* I lived in the southern regions of the country*,* where there are diverse ethnicities*,* beliefs*,* and cultures; even different languages matter in conveying ideas. So*,* we selected local individuals from each region and trained them as health workers*,* which proved to be a very successful strategy.”*

#### Providing necessary facilities in the form of a health network to enhance equitable access

One of the innovative steps in the healthcare field that has led to significant national achievements was the creation of a health network with multiple levels of service delivery and a referral system. This equity-oriented approach to PHC, summarised as the PHC and DHNs, became a cornerstone of healthcare provision.

One participant remarked: P 7: *“When discussing the structure and technical foundations necessary to establish DHNs*,* we must consider the chain of health services based on the population’s needs and financial capabilities*,* as defined by this system. The creation of a DHN structure and facilities at the PHC level in 1985–1986 was a pivotal milestone.”*

### Process

#### Putting on the agenda and formulating policies

The interviewees highlighted that prioritising healthcare delivery by non-physician personnel, with a focus on prevention rather than treatment, was a key factor that contributed to the inclusion of these policies in the national agenda. Another driving factor was the high mortality rate of children under five years of age due to preventable causes. Additionally, participants pointed to the government’s favourable stance on healthcare issues and its aim to reduce long-term healthcare costs as reasons for the policy’s adoption.

One participant noted: P 6: *“In the new government*,* attention shifted to the quality of health services and related expenses. I believe our professional perspectives deepened*,* and we aimed to focus more on health alongside treatment.”*

#### Policy implementation

Both high-level decision-makers and experts working within the PHC system suggested that the success of policies in achieving health equity was influenced by how they were implemented, as well as by the various circumstances encountered during their execution. These participants indicated that while Iran’s socioeconomic, health, and demographic conditions evolved during the implementation of the PHC and DHNs policies, the authorities responsible for healthcare did not adapt the system’s structure or service capacity in response to these changes.

One interviewee commented: P 2: *“The socioeconomic and health conditions of the country have changed—epidemiological trends*,* disease burden*,* resource distribution*,* infrastructure*,* mortality patterns*,* population structure*,* urbanisation*,* and people’s lifestyles. However*,* authorities did not update DHNs to accommodate the growing needs of populations with NCDs. As a result*,* rural areas may have limited access to NCD care or treatment for advanced NCDs.”*

#### Evaluation

Several interviewees expressed concerns over the absence of rigorous monitoring and evaluation mechanisms for the PHC and DHNs policies and their outcomes. Some suggested that the establishment of electronic infrastructures would have facilitated the monitoring and evaluation processes.

As one participant remarked: P 16: *“We must always adhere strictly to the guidelines. The PHC*,* the DHNs*,* staff performance*,* coordination across different levels of the DHNs*,* and the referral system should have been continuously evaluated*,* with any shortcomings promptly addressed.”*

### Key actors

Based on participants’ views, initially, there were two main groups that opposed the PHC and DHNs policies: doctors and other healthcare providers in the private sector. Their opposition stemmed primarily from the health system’s increased emphasis on preventive care, which reduced the number of general practitioner (GP) and specialist doctor visits due to the establishment of a referral system. In contrast, other key stakeholders, such as the Ministry of Health and Medical Education (MoHME), the Ministry of Cooperatives, Labour, and Social Welfare (MoCLSW), the Social Security Organization (SSO), the Relief Foundation, local institutions, and international organisations, generally supported these policies.

However, the level of support from these actors evolved over time. Approximately two decades after the DHNs establishment, the MoHME, which had been a primary advocate for the DHNs, showed diminished support. The ministry’s focus shifted towards investment in treatment and secondary care, rather than maintaining the centrality of primary healthcare.

As one participant noted: P 11: *“The main role players are the government*,* the parliament*,* the MoHME*,* provincial medical universitiess*,* international organisations such as the WHO*,* and local institutions such as village heads. The most important actors are the people in deprived areas*,* whether they are receiving services or serving as doctors*,* dentists*,* midwives*,* disease control specialists*,* nurses*,* or health workers. However*,* the Ministry [of Health and Medical Education] has shown diluted support [towards PHC] over the last 20 years. They now seem more focused on earning revenue from treatment.”*

### Success factors

Participants agreed that the PHC and DHNs policies were initially successful, particularly in improving health equity indicators related to communicable diseases, maternal and newborn mortality, and morbidity. The factors contributing to this success, as identified by participants, are outlined below:

#### Appropriate equity-based design: regionalisation, service integration, and service delivery through the referral system

PHC serves as the first point of contact for individuals and families within the healthcare system, facilitating access to more specialised services as needed. A well-planned PHC system, based on clinical guidelines and the principles of horizontal and vertical equity, helps ensure equitable access to healthcare services. It also promotes the redistribution of health resources across various service levels and regions, ensuring continuity of care and equity in service delivery.

As one participant remarked: P 8: *“The experiences of different countries*,* including ours*,* show that if we have a powerful PHC*,* we can solve many of our problems. The experiences of countries like Thailand*,* England*,* and many OECD nations showed that if they designed PHC well*,* they could improve equity and access.”*

#### Strict rules

As discussed in the policy context, the existence of robust laws, reflected in foundational documents such as the Constitution, national development plans, public insurance laws, and Council of Ministers’ approval letters, played a critical role in advancing PHC, DHNs, and referral system policies. These regulations not only supported the efficient implementation of these policies but also helped curtail the provision of unnecessary services, reducing costs for patients, payers, and the government, thus promoting equity.

One participant highlighted: P 17: *“Since the mid- 50 s*,* the issues related to the implementation of PHC and its support have been specified very well and precisely in our laws*,* and even before that*,* the establishment of the DHNs in the laws was sometimes discussed. The regulations could stop the delivery of unnecessary services and save lots of money for people*,* payers*,* and the government. This is equity.”*

P 11: *“I remember a time when the government’s priority*,* aside from the war [against Iraq]*,* was establishing Health Houses and Health Posts and training a workforce for them. All medical universities were under the Ministry’s monitoring and scrutiny to develop DHNs even those had same border [with Iraq].”*

### Failure factors

Although the PHC and DHNs policies initially achieved some targets, participants noted that they ultimately failed to sustain their mission of achieving long-term health equity. The factors contributing to this failure are discussed below:

#### Decreased support of the referral system

Several years after the implementation of the PHC and DHNs policies, the referral system faced significant disruption due to various factors: a lack of trust in the quality of primary-level services due to populations’ increased expectations, insufficient skills among personnel in communicating with the public who were then more knowledgeable, public’s poor awareness of the services available within the referral system, inadequate use of media to promote the importance of primary care, a lack of a self-care culture, and a consumer preference for costly specialised services. Additionally, conflicts between general practitioners, specialists, and the Ministry of Health and Medical Education (MoHME) exacerbated these issues. The collapse of the referral system resulted in unequal access to healthcare, favouring those with better financial means and geographical access.

One participant explained: P 14: *“The managers of the MoHME did not make their standards objective*,* or they could not defend against pressure groups*,* such as the Parliament*,* governors*,* representatives*,* doctors*,* etc. Also*,* people liked to refer to doctors directly rather than following referral routes. Therefore*,* the referral system was not supported as it was initially.”*

#### Lack of participation and common belief among policymakers, executives, and people and conflicts of interest

The extent of intra- and inter-sectoral participation within the health system, necessary to realise the PHC and DHNs’ goals, was not clearly or effectively defined in the years following its implementation. Conflicts of interest among key stakeholders, particularly within the health sector, distorted the advancement of the DHNs and referral systems. Some physicians, for instance, opposed the referral system due to concerns about losing their patients and income. Additionally, the blurred boundaries between the public and private sectors, along with the promotion of fee-for-service payments, further fuelled these conflicts of interest.

As one participant stated: P 23: *“The problem is that we even considered the conflict of interest; for example*,* the concern of specialists about the reduction of their patients and so their income. At the macro level*,* even insurance organisations and the Ministry [of Health and Medical Education] sometimes were against each other. Such trends cause more difficult access to the deprived population due to the higher charges getting imposed on these groups*,* especially when doctors take their patients from the public sector to private hospitals.”*

Moreover, many people, particularly in urban areas, remain unaware of the concept of the referral system and the necessity of adhering to it. No significant efforts have been made to develop an intellectual infrastructure to educate individuals and families about the importance of prevention, self-care, and health-related expenditures.

#### Transition from communicable diseases to NCDs and the lack of alternative plans

Many experts, especially those within provincial medical universities and health purchasing organisations, noted that while the PHC and DHNs policies were initially well-designed, they have failed to adapt to evolving health challenges, particularly the transition from communicable diseases to NCDs. The system has not been updated to reflect the changing burden of disease or to incorporate new technologies, leaving it unable to effectively address NCDs, which now represent a significant health burden in Iran. This failure to adapt has undermined the overall function of the PHC and DHNs.

One participant highlighted this issue: P 12: *“In the 1990 s*,* the face of death changed. We encountered the phenomenon of the iceberg in 29 provinces and saw many diseases transmitted through drinking water and food; these diseases have changed to NCDs*,* such as mental health problems and ischemia. When we saw this*,* we didn’t have the model to intervene in society. The change of lifestyle was not penetrated in our PHC. Payers do not pay for a healthy lifestyle. They generally do not support screening or preventive care. However*,* the private sector is now active in giving healthcare to populations with higher financial status*,* although through invasive or semi-invasive procedures.”*

## Discussion

**Fig. 2:**
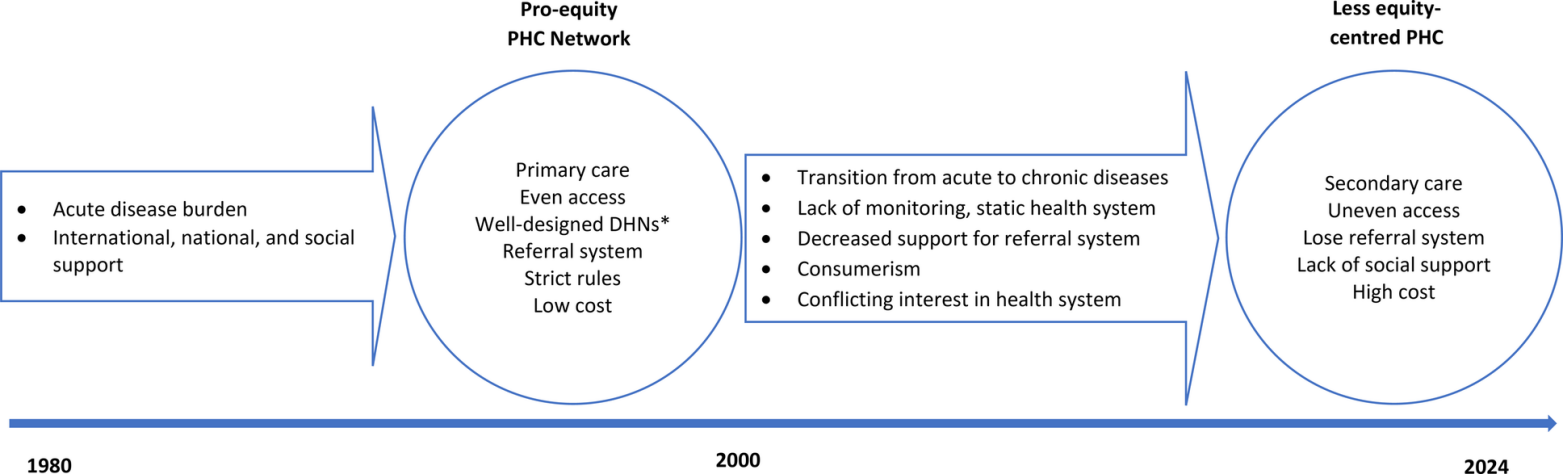
The timeline of Iranian PHC policies and its influence on health equity *: District-level Health Networks

This study aimed to explore how Primary Health Care (PHC) policies in Iran have influenced equity within the health system. We commenced with a review and screening of PHC policies, focusing specifically on their equity implications, followed by interviews with experts and policymakers in the areas of the PHC and health equity. Our historical analysis, framed by the approach suggested by Walt and Gilson [22], reveals a significant shift in Iranian PHC policies over the past fifty years, from a strong emphasis on equity to a less equity-centred focus. Figure 2 illustrates the timeline of this shift.

As detailed in the “Results” section, the Iranian PHC policies, initiated in the 1980 s, were driven by the urgent need to reduce high rates of mortality and morbidity associated with communicable diseases, as well as maternal and neonatal health issues, particularly in rural and deprived regions. With both international and social support, the government successfully established PHC and DHN policies aimed at ensuring equitable access to primary care. These policies included low-cost interventions regulated by a strict referral system designed to prevent unnecessary and costly healthcare.

These initial initiatives led to significant improvements in health indicators and equity outcomes [16]. However, this focus on equity began to diminish after approximately two decades, resulting in a gradual shift away from the original goals of PHC and DHNs.

The transition of the burden from acute and communicable diseases to chronic and non-communicable diseases (NCDs) necessitates the adaptation of the PHC and DHNs to support lifestyle changes among the population. However, this adaptation was not adequately addressed, and the Ministry of Health and Medical Education (MoHME) did not effectively monitor this shift in health needs (Process). This lack of adaptation limited access to the preventive care against NCDs, resulting in these conditions often being managed only in their advanced stages. At this point, treatment was generally less accessible, both financially and geographically, particularly for people living in rural or disadvantaged areas.

Similarly, the literature indicates that many countries, particularly developing ones, face challenges in integrating NCD services into their PHC systems. A review across African nations found that no country met al.l the criteria suggested by the WHO for integrating NCD services into PHC [24]. Another review highlighted that many low- and middle-income countries (LMICs) lacked sufficient government support, infrastructure, and monitoring systems, indicating that their readiness for such reforms was inadequate [5]. Studies conducted in Ethiopia and Bangladesh identified key barriers to integrating NCD services into PHC, including insufficient political commitment, weak governance, poor intersectoral coordination, and inadequate funding [25, 26], all of which align with our findings. Consequently, Iran’s health system was unprepared to integrate NCD management into the PHC framework, resulting in chronic diseases being addressed only at later stages and predominantly within secondary healthcare facilities.

This shift also coincided with a consumerist health culture among the public, favoring treatment over prevention. Due to the lack of social support for the referral system and a public preference for transitioning from low-cost primary care to more expensive secondary care, the MoHME increasingly focused its policies on secondary care, such as the establishment of additional hospitals. International experiences demonstrate that governments can effectively strengthen referral systems and gatekeeping when supported by public trust and engagement [27]. In contrast consumer-driven attitudes towards healthcare, where people seek tests, treatments, or specialist consultations without clear necessity, can make it harder for governments to prioritise the essential services and build effective primary care models. However, populations require support to make informed choices, which includes addressing barriers such as low health literacy, cultural norms, and limited access to educational resources. This highlights the shared responsibility between governments and populations in promoting primary care utilisation, which has not been the case in Iran [28].

Additionally, the dominance of fee-for-service payment structures in provider income (both in the public and private sectors) and the blurred boundaries between these sectors have led to simultaneous engagements by doctors in both settings, undermining the robust support for the PHC and DHNs (Actors). Evidence suggests that conflicts of interest can be inherent in governmental attempts to establish preventive policies for NCDs [29]. Moreover, the risk of conflicting interests has been reported among countries in the Eastern Mediterranean Region as they strive to utilise both public and private sectors for integrating NCDs into PHC [30].

Such trends have led to the weakening of the initially established DHNs and referral routes within Iran’s health system. As a result, populations with better financial status have significantly greater access to care and services, even within the public sector. The literature supports our findings. A study in China demonstrated that establishing referral systems and gatekeeping can improve equity of access, while their removal can exacerbate inequities [5]. Additionally, research in El Salvador indicated that the government’s lack of preventive programs for NCDs through PHC results in costly interventions that are not accessible to poorer populations [31].

Our study had some limitations and strengths. We were unable to incorporate quantitative data as a triangulation method to support or validate the qualitative findings due to time and funding limitations. However, our broad view on policies over half a century provided a comprehensive analysis and understanding of the Iranian health system and its PHC policies. Additionally, our study benefited from the involvement of key participants with significant experience and knowledge in the areas of health policy and equity. Future research can test our findings based on quantitative approach.

## Conclusion

Considering the achievements and failures of PHC policies in Iran in achieving persistent equity, several implications can be drawn. Firstly, PHC policies should be adaptive to emerging health concerns and shifts in disease burden caused by various factors such as advancements in health and technology, lifestyle changes, and epidemiological transitions. Equity considerations necessitate the integration of NCD care into PHC, ensuring that all people have easy and affordable access to care. However, such reforms to integrate NCDs into the PHC package can be challenging for governments. Healthcare policymakers should conduct ongoing monitoring of health issues and disease trends at both global and national/regional levels.

After acknowledging the need for PHC reform, governments should actively support it and ensure political will is sustained. Both the public and healthcare staff must be trained accordingly. Populations should be encouraged to pursue preventive care for NCDs and adopt healthier lifestyles, while healthcare staff need training in new screening and education techniques. Through these initiatives, as populations prioritize preventive health over treatment and respect referral routes, achieving health equity becomes more feasible. Furthermore, addressing barriers—such as the motivations for neglecting PHC among some stakeholders—will be crucial. It is important to note that without effective integration of NCD care into PHC, populations with lower financial or cultural access will disproportionately suffer from NCDs, facing costly and long-term consequences.

## Supplementary Information

Supplementary Material 1.

## Data Availability

The quantitative data and the transcripts of the anonymised interviews in Persian are available from the corresponding author on reasonable request.
