# Supplementary material for: How Iranian primary health care policies influenced equity: a historical critical analysis from policymakers’ perspectives
Source: BMC Health Serv Res. 2025 Nov 25;25:1527. doi: 10.1186/s12913-025-12736-3 (PMC12648947; doi:10.1186/s12913-025-12736-3)
Supplement: Supplementary file 1 — Supplementary Material 1. [file 12913_2025_12736_MOESM1_ESM.docx]

**Appendix**

Topic guide of semi-structured interview

Code: ………………. Place of interview: ………………… Date of interview: …………...

Selective Policies Relative to Primary Health Care (PHC), Health Network, and Referral System

**Part 1. The demographic characteristics of participants**

Gender: ………………………. Name(optional): ……………………

Qualification: ……………………… Age: ……………. years

Position: ………………………. Field of study: ………………………………

Years of experience: …………………………. Organization: ………………………….

**Part 2. Questions**

- What is your opinion on the policies related to Primary Health Care (PHC), network, and referral system that have been selected for this study? In general, what are your thoughts on these policies?

- What is the content of these policies in your opinion? Do you have any comments on their content?

- What was the main purpose of proposing and implementing these policies related to PHC, network, and referral system? How did these policies come to be on the agenda? Has equity been considered as one of the primary goals of these policies? If so, how?

- Do you know which groups were involved in formulating these policies?

- In your opinion, which laws, and upstream and downstream documents have supported the implementation of these policies? Has any legal infrastructure been established to facilitate and consolidate the implementation of these policies?

- Under what circumstances were these policies adopted and what were the primary challenges or problems at that time? Did socio-cultural, economic, or political conditions play a role in their implementation? What kind of internal and external cooperation is needed to facilitate the implementation of policies related to equity, especially those related to PHC, network, and referral system?

- In your opinion, have these policies been successful in ensuring health equity in society? Which dimensions of equity have been affected by these policies? Please provide an example.

- Who were the stakeholders involved in creating, establishing, and promoting these policies? What was their role in promoting or hindering health equity?

- What is your opinion on the reasons for the success or failure of these policies? What were the main contributing factors?

- What solutions do you suggest increasing equity in policies related to PHC, networks, and referral system? Please explain your thoughts briefly.

- Do you have anything else to add? If there is something we have not asked for, please let us know.

- Can you recommend an expert in this field whose input would be valuable for this study?
